# Supplementary material for: The ANGPTL3-4-8 Axis in Normal Gestation and in Gestational Diabetes, and Its Potential Involvement in Fetal Growth
Source: Int J Mol Sci. 2023 Jan 27;24(3):2486. doi: 10.3390/ijms24032486 (PMC9917010; doi:10.3390/ijms24032486)
Supplement: Supplementary file 1 [file ijms-24-02486-s001.zip › Supplementary Material.pdf]

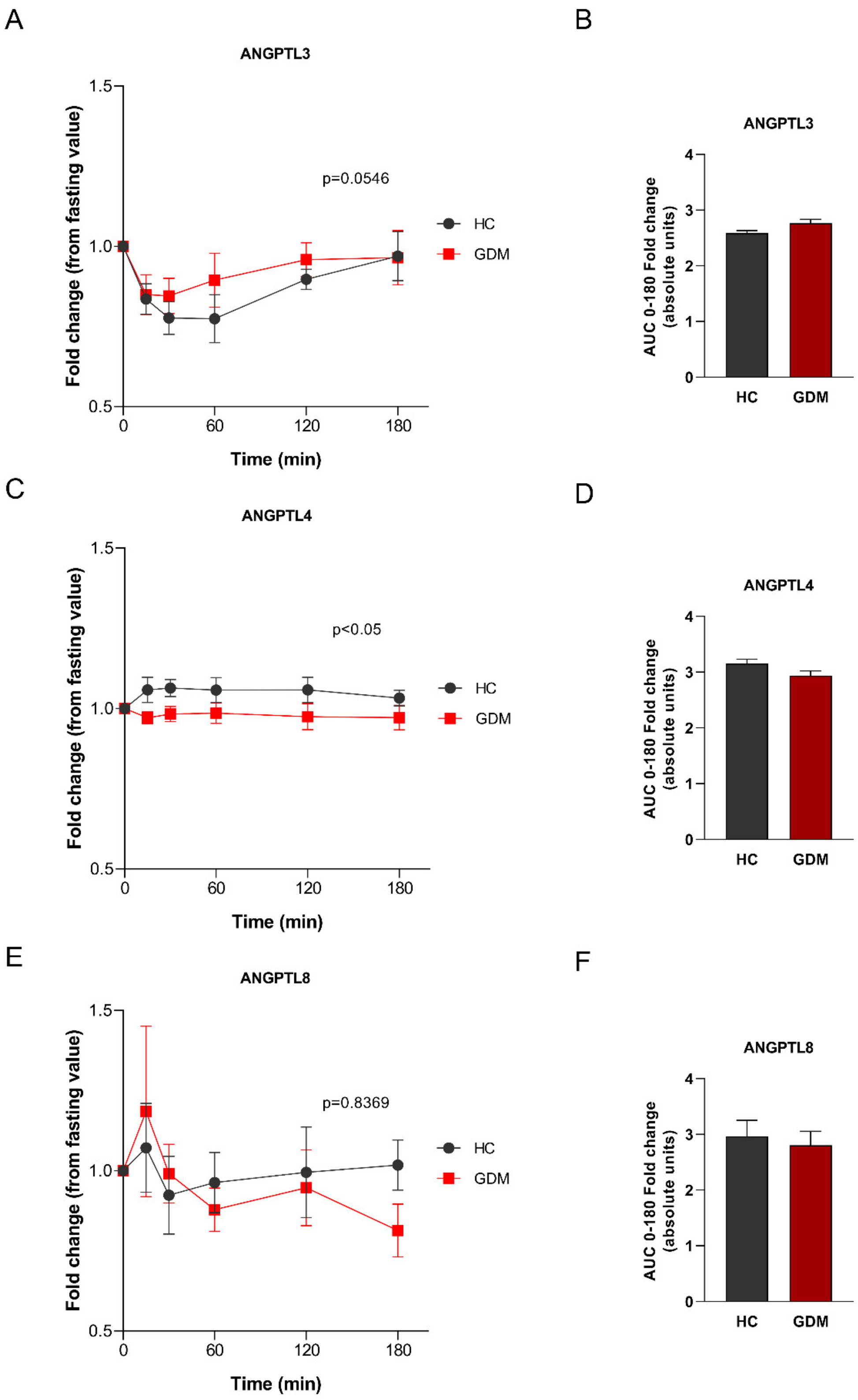

A
ANGPTL3
Fold change (from fasting value)
1.5
1.0
0.5
p=0.0546
HC
GDM
0
60
120
180
Time (min)
B
ANGPTL3
AUC 0-180 Fold change (absolute units)
4
3
2
1
0
HC
GDM
C
ANGPTL4
Fold change (from fasting value)
1.5
1.0
0.5
p<0.05
HC
GDM
0
60
120
180
Time (min)
D
ANGPTL4
AUC 0-180 Fold change (absolute units)
4
3
2
1
0
HC
GDM
E
ANGPTL8
Fold change (from fasting value)
1.5
1.0
0.5
p=0.8369
HC
GDM
0
60
120
180
Time (min)
F
ANGPTL8
AUC 0-180 Fold change (absolute units)
4
3
2
1
0
HC
GDM

**Figure S1: ANGPTL3, 4, and 8 responses to the meal test in healthy control women versus GDM women.** This figure shows time curves of serum ANGPTL3 (A), ANGPTL4 (C), and ANGPTL8 (E) response to a Meal test (fold increase over basal values). The figure also shows the Area Under the Curve of the ANGPTL3 (B), ANGPTL4 (D), and ANGPTL8 (F). Data are mean ± S.E.M. AUC differences were assessed by Kruskal-Wallis test followed by Dunnett's correction, and time curves were compared using repeated measures ANOVA with Bonferroni post hoc test (*P* values refer to curve differences between healthy control and GDM women). HC: healthy control; GDM: gestational diabetes; ANGPTL3: Angiopoietin-Like-Protein 3; ANGPTL4: Human Angiopoietin-Like Protein 4; ANGPTL8: Human Angiopoietin-Like Protein 8.

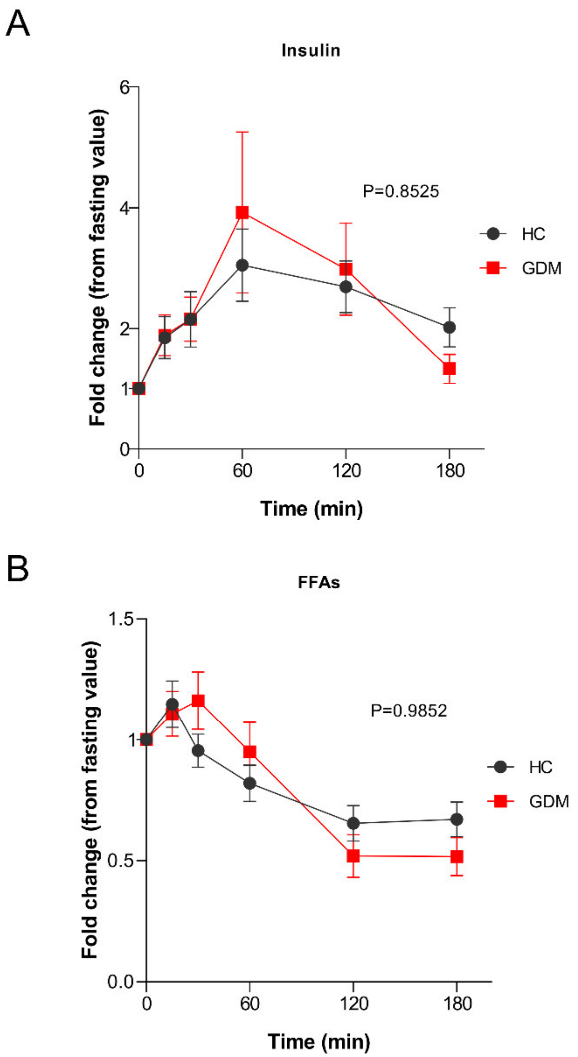

A
Insulin
P=0.8525
HC
GDM
Fold change (from fasting value)
Time (min)
0
1
2
4
6
0
60
120
180
B
FFAs
P=0.9852
HC
GDM
Fold change (from fasting value)
Time (min)
0.0
0.5
1
1.5
0
60
120
180
C
Glucose
P=0.4317
HC
GDM
Fold change (from fasting value)
Time (min)
0.8
1
1.3
1.5
0
60
120
180
D
Triglycerides
P=0.2098
HC
GDM
Fold change (from fasting value)
Time (min)
0.8
1
1.2
0
60
120
180

**Figure S2: Serum insulin, glucose, free fatty acids and triglyceride response to the meal test in healthy control versus GDM women**. The figure shows time curves of serum Insulin, glucose, free fatty acids (FFA) and triglycerides response during a Meal test (fold increase over basal values). Data are mean ± S.E.M. repeated measures ANOVA with Bonferroni post hoc test (*p* values refer to curve differences between healthy control and GDM women). HC: healthy control; GDM: gestational diabetes; ANGPTL3: Angiopoietin-Like-Protein 3; ANGPTL4: Human Angiopoietin-Like Protein 4; ANGPTL8: Human Angiopoietin-Like Protein 8; FFAs: Free Fatty Acids; TGs: Triglycerides.

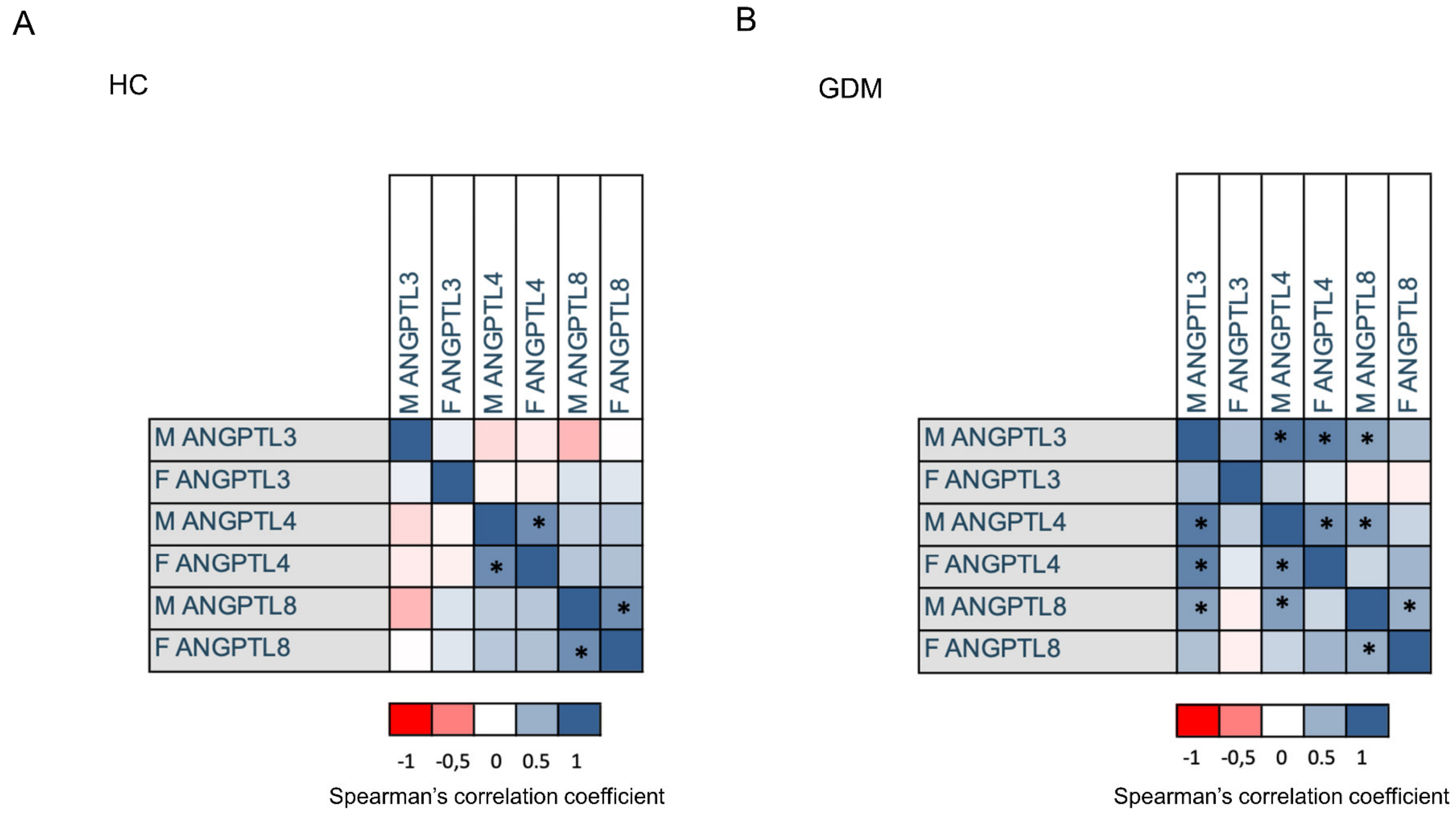
A
HC
B
GDM
M ANGPTL3
F ANGPTL3
M ANGPTL4
F ANGPTL4
M ANGPTL8
F ANGPTL8
-1
-0,5
0
0.5
1
Spearman's correlation coefficient

**Figure S3. The relationship between the expression of the different ANGPTLs at the same location, and between the different locations, is modified by GDM.** Heatmap of the associations ANGPTL3, 4, and 8 at the same location and different locations in the healthy control (A) and the GDM group (B). Gene expression data are expressed as fold change. Correlations were calculated using Spearman's correlation coefficient. **P*<0.05. HC: healthy control; GDM: gestational diabetes mellitus; M before the name of any gene expression indicates expression on the maternal side of the placenta; F before the name of any gene expression indicates expression on the fetal side of the placenta. ANGPTL3: Angiopoietin-Like-Protein 3; ANGPTL4: Human Angiopoietin-Like Protein 4; ANGPTL8: Human Angiopoietin-Like Protein 8.

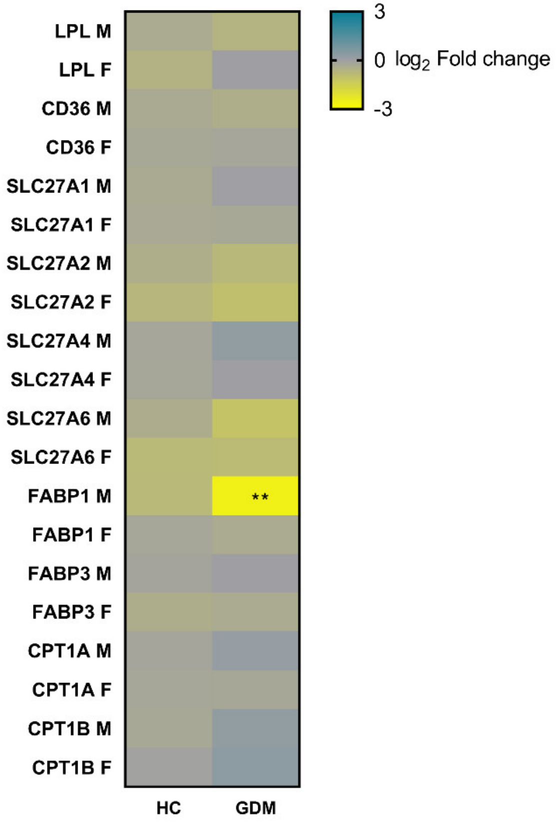

LPL M
LPL F
CD36 M
CD36 F
SLC27A1 M
SLC27A1 F
SLC27A2 M
SLC27A2 F
SLC27A4 M
SLC27A4 F
SLC27A6 M
SLC27A6 F
FABP1 M
FABP1 F
FABP3 M
FABP3 F
CPT1A M
CPT1A F
CPT1B M
CPT1B F
**
HC
GDM
3
0
-3
$log_2$ Fold change

**Figure S4**. **Comparison of gene expression of LPL and proteins involved in lipid transport in healthy control women versus GDM women**. (A) Heatmap comparing lipoprotein lipase and lipid transport gene expression between the maternal and fetal side of the placenta (left panel) in healthy control women (N=19 in each), and (right panel) in the gestational diabetes group (N=20). (B) Heatmap comparing ANGTPL3, 4, and 8 expression on both sides of the placenta according to glucose tolerance status (HC vs GDM**).** Differences assessed by two-way ANOVA multiple comparison test followed by Bonferroni correction. Data are normalized to the mean of the control group for each gene and side and are shown as mean ± S.E.M. **$P<0.01$. HC: healthy control; GDM: gestational diabetes; M before the name of any gene expression indicates expression on the maternal side of the placenta; F before the name of any gene expression indicates expression on the fetal side of the placenta.LPL: Lipoprotein Lipase, CD36: Cluster of Differentiation 36; SLC27A1, SLC27A2, SLC27A4, SLC27A6: Solute Carrier Family 27 Member 1, 2, 4 and 6, respectively; FABP1, FABP3: Fatty Acid Binding Protein 1 and 3, respectively; CPT1A, CPT1B: Carnitine Palmitoyltransferase 1A and 1B, respectively.
